# A study of antibiotic resistance pattern of clinical bacterial pathogens isolated from patients in a tertiary care hospital

**DOI:** 10.3389/fmicb.2024.1383989

**Published:** 2024-04-17

**Authors:** Vishal L. Handa, Bhoomi N. Patel, Dr. Arpita Bhattacharya, Ramesh K. Kothari, Dr. Ghanshyam Kavathia, B. R. M. Vyas

**Affiliations:** ^1^Department of Biosciences, Saurashtra University, Rajkot, Gujarat, India; ^2^Department of Microbiology, Pandit Deendayal Upadhyay Medical College, Rajkot, Gujarat, India

**Keywords:** antimicrobial resistance, extended spectrum-**β**-lactamase, methicillin-resistant *Staphylococcus aureus*, metallo-**β**-lactamase, erythromycin-induced clindamycin resistance

## Abstract

We investigated antibiotic resistance pattern in clinical bacterial pathogens isolated from in-patients and out-patients, and compared it with non-clinical bacterial isolates. 475 bacterial strains isolated from patients were examined for antibiotic resistance. *Staphylococcus* spp. (148; 31.1%) were found to be the most prevalent, followed by *Klebsiella pneumoniae* (135; 28.4%), *Escherichia coli* (74; 15.5%), *Pseudomonas aeruginosa* (65; 13.6%), *Enterobacter* spp. (28; 5.8%), and *Acinetobacter* spp. (25; 5.2%). Drug-resistant bacteria isolated were extended spectrum-β-lactamase *K*. *pneumoniae* (8.8%), *E*. *coli* (20%), metallo-β-lactamase *P*. *aeruginosa* (14; 2.9%), erythromycin-inducing clindamycin resistant (7.4%), and methicillin-resistant *Staphylococcus* species (21.6%). Pathogens belonging to the Enterobacteriaceae family were observed to undergo directional selection developing resistance against antibiotics ciprofloxacin, piperacillin-tazobactam, cefepime, and cefuroxime. Pathogens in the surgical ward exhibited higher levels of antibiotic resistance, while non-clinical *P*. *aeruginosa* and *K*. *pneumoniae* strains were more antibiotic-susceptible. Our research assisted in identifying the drugs that can be used to control infections caused by antimicrobial resistant bacteria in the population and in monitoring the prevalence of drug-resistant bacterial pathogens.

## 1 Introduction

Development and spread of antimicrobial resistance (AMR) in pathogenic bacteria is a global problem. According to GBD, 33 bacterial diseases were responsible for 7.7 million deaths worldwide (Ikuta et al., 2022). The proportion of animals infected with drug-resistant bacteria increased by 50% during 2000–2018, limiting the number of available treatments (Van Boeckel et al., 2019). Antibiotics used for treating lower respiratory tract infections in children grew by 46% globally during 2000–2018 (Browne et al., 2021). The top six antibiotic-resistant bacteria causing human deaths in the United States of America are *Escherichia coli*, *Streptococcus pneumoniae*, *Staphylococcus aureus*, *Acinetobacter baumannii*, *Klebsiella pneumoniae*, and *Pseudomonas aeruginosa* (CDC, 2019). According to the United States Center for Disease Control and Prevention, there are 2.8 million cases of infections and 35,000 deaths caused by antibiotic resistant bacteria yearly, in the country. The extended spectrum-β-lactamase (ESBL) Enterobacteriaceae, methicillin-resistant *Staphylococcus aureus* (MRSA), vancomycin-resistant *Enterococcus*, and drug-resistant *Mycobacterium tuberculosis* are considered severe threats to human lives (CDC, 2019). MRSA-related death rate of 39.1% in middle-income and 32.1% in high-income countries, was reported by Bai et al. (2022). Natural selection facilitates the evolution of antibiotic resistance in bacteria especially in antibiotic-contaminated aquatic environments, which serve as routes for the spread of resistant bacteria to livestock, poultry, humans, and other animals (Baquero et al., 2021; Larsson and Flach, 2022). High antibiotic use, fixed-dose combinations, self-medication, access to antibiotics without a prescription from a doctor, poor management of industrial effluent treatment plants, lack of hygenic condition, and inefficient infection control procedures in healthcare, are some of the factors contributing to India’s high AMR proportions (Gandra et al., 2017). Carbapenem and colistin-resistant *K*. *pneumoniae* was responsible for 69% death rate in India during 2011–2015 (Kaur et al., 2017). Bacteria isolated from soil and water samples near pharmaceutical industrial areas in Hyderabad revealed up to 70% resistance against cephalosporin antibiotics (Britto et al., 2019). In 2015, the cephalosporins were most frequently used in India, followed by penicillins and fluoroquinolones; majority of pathogenic bacteria isolated were resistant to cephalosporins, followed by fluoroquinolones and penicillins (Klein et al., 2019). *Escherichia coli* isolated from domestic (25%) and hospital wastes (95%) were observed to be resistant to third-generation cephalosporins (Akiba et al., 2015).

In this study, we aimed to provide descriptive data on infections and patterns of antibiotic resistance of the top six bacterial pathogens in Pandit Deendayal Upadhyay (PDU) Medical College and Hospital Rajkot, Gujarat. We looked for an answer to the crucial medical query “Does the pattern of antibiotic resistance of bacterial infections vary with isolation sources?” We also intended to compare the prevailing patterns of antibiotic resistance in clinical and non-clinical strains of *P*. *aeruginosa* and *K*. *pneumoniae*. We also focused on erythromycin-induced clindamycin resistance (EICR) and MRSA in *Staphylococcus* species and ESBL and metallo-β-lactamase (MBL) in Gram-negative bacteria.

## 2 Materials and methods

### 2.1 Location and context of the study

The PDU Medical College/Hospital is a tertiary care and teaching hospital that provides a full range of health care services, including medical, surgical, and superspecialty services, to patients in and around Rajkot district. In a 100-km radius of Rajkot, there is only one multispeciality government hospital. Every day, more than 400 patients load from Rajkot city as well as rural areas of Rajkot district. Also, people from Amreli, Jamnagar, Junagadh, Kachchh, Morbi, Porbandar, Surendranagar, and Veraval visit PDU Medical College/Hospital Rajkot for treatments. PDU Medical College/Hospital is located in the center of the Saurashtra region in Rajkot, Gujarat.

### 2.2 Sample collection and isolation of pathogens

Based on the data of the past 6 months, we selected the six most commonly reported pathogens from hospitalized and outpatient specimens, including *Pseudomonas aeruginosa*, *Escherichia coli*, *Enterobacter* spp., *Klebsiella pneumoniae*, *Staphylococcus* spp., and *Acinetobacter* spp. The top six most frequently observed bacterial strains were selected for the present study. Samples were collected during March to June 2022 from the in-patients and out-patients at PDU Medical College/Hospital in Rajkot, Gujarat. Samples were collected in sterile containers according to Cheesbrough (2005) in various wards by the assigned clinicians, and they were processed further at the bacteriology lab immediately. Preservation and storage of specimens varied from place to place and time to time; for example, blood, urine, and sputum were stored until the analysis and discussion with the assigned doctor. While for precious specimens like postoperative samples, body fluids were stored for 7–10 days, etc. Gender, age, ward, collection date, specimens, and other information were noted along with the sample collection. Ten samples were randomly selected daily for this survey, conducted for 3 months. PEEKSA (*Pseudomonas aeruginosa*, *Escherichia coli*, *Enterobacter* spp., *Klebsiella pneumoniae*, *Staphylococcus* spp. and *Acinetobacter* spp) were isolated from the collected samples (CLSI, 2018). Blood samples collected from adult patients (10–20 mL) and pediatric patients (5–10 mL) were mixed in brain heart infusion broth bottles and analyzed for bacterial growth up to 7 days using automated blood culture system BD-BACTEC FX40 (United States) (Procop et al., 2020). Absence of turbidity after 7 days was considered negative. Positive samples were subcultured on blood agar, nutrient agar, and MacConkey agar plates. Urine samples (20–30 mL) were collected in a sterile plastic container (50 mL capacity) and streaked on MacConkey agar, blood agar, cysteine lactose electrolyte deficient agar, and nutrient agar plates. Pus/swab samples were streaked on nutrient agar, blood agar, and MacConkey agar plates. Sputum samples (2–5 mL) were collected in a sterile plastic container (50 mL capacity) and streaked on chocolate agar, blood agar, nutrient agar, and MacConkey agar plates. The samples except blood were processed the same day; streaked plates were incubated at 35°C for 24–72 h and subcultured on nutrient agar plates. Bacterial identification was done based on colony morphology, biochemical tests, and the Gram reaction (Collee et al., 1996; Procop et al., 2020).

### 2.3 Antibiotic susceptibility test of bacterial isolates

Gram-positive and-negative bacterial isolates were evaluated for antibiotic susceptibility employing Kirby-Bauer disk-diffusion method (CLSI, 2018). A single colony was picked and suspended in sterile normal saline (0.85% NaCl) to generate the equivalent of 0.5 McFarland standard solution. 1 mL of bacterial suspension was mixed with 19 mL of sterile Muller-Hinton soft agar (45°C) and poured in Petri plates, incubated at 35°C for 24 h after the transfer of antibiotics disks by disk dispenser. PEEKSA isolates were classified as resistant, intermediate, and sensitive to antibiotics on the basis of the size of the zone of inhibition according to the Clinical Laboratory Standard Institute (CLSI) guidelines. Antibiotics used for antibiotic susceptibility test of *Enterobacter* spp., *Klebsiella pneumoniae*, and *Escherichia coli* isolated from pus, swabs, sputum, and blood samples were ciprofloxacin (CIP), levofloxacin (LVX), gentamicin (GM), amikacin (AN), meropenem (MEM), cefuroxime (CXM), cefotaxime (CTX), ceftazidime (CAZ), ceftazidime-clavulanate (CAC), cefepime (FEP), piperacillin-tazobactam (TZP), trimethoprim-sulfamethoxazole (SXT), tetracycline (TE), and ampicillin-sulbactam (SAM). Strains isolated from urine samples were also evaluated for sensitivity against the antibiotics TR, NX, and NIT along with the above-mentioned antibiotics. Imipenem-EDTA (IE), imipenem (IPM), aztreonam (ATM), CIP, GM, LVX, AN, and FEP were employed for the evaluation of the antibiotic sensitivity of *Pseudomonas aeruginosa*. *Staphylococcus* spp. were against erythromycin (E), linezolid (LZD), CIP, rifamycin (RIF), clindamycin (CM), TE, vancomycin (VA), SXT, GM, chloramphenicol (C), meropenem (MEM), cefoxitin (FOX), and penicillin (P). Antibiotics class, abbreviation, and concentration in Supplementary Table S1.

### 2.4 Data processing and analysis

According to the CLSI recommendations, bacterial pathogens were classified as sensitive, intermediate, and resistant based on antibiotic susceptibility values (CLSI, 2022). Descriptive statistics such as relative abundance, percentage of categorical variation, and frequency were calculated. The chi-square test was used to compare the abundance of bacterial isolates with patient specimens. A Tukey *post-hoc* test was conducted for multiple comparisons between the mean values of the number of resistant antibiotics. *p* values less than 0.05 were considered statistically significant. Antibiotic resistance index (R_I_) was derived by *n*/*N* where, “*n*” is number of resisrant isolates and “*N*” is the total number of isolates tested. Pearson’s correlation analysis was studied between isolation source and its antibiotic resistance pattern (antibiotics that are tested more than 90% were used for the correlation study). Natural selection was determined by (*n*/*N*) × 100, where “n” is the number of sensitive, intermediate, or resistant phenotypes expressed by each isolate and “*N*” is the total number of bacterial isolates.

## 3 Results

475 pathogenic bacterial strains isolated from 910 clinical samples were investigated for their antibiotic resistance patterns.

### 3.1 Prevalence of bacterial pathogens in clinical samples

**Figure 1:**
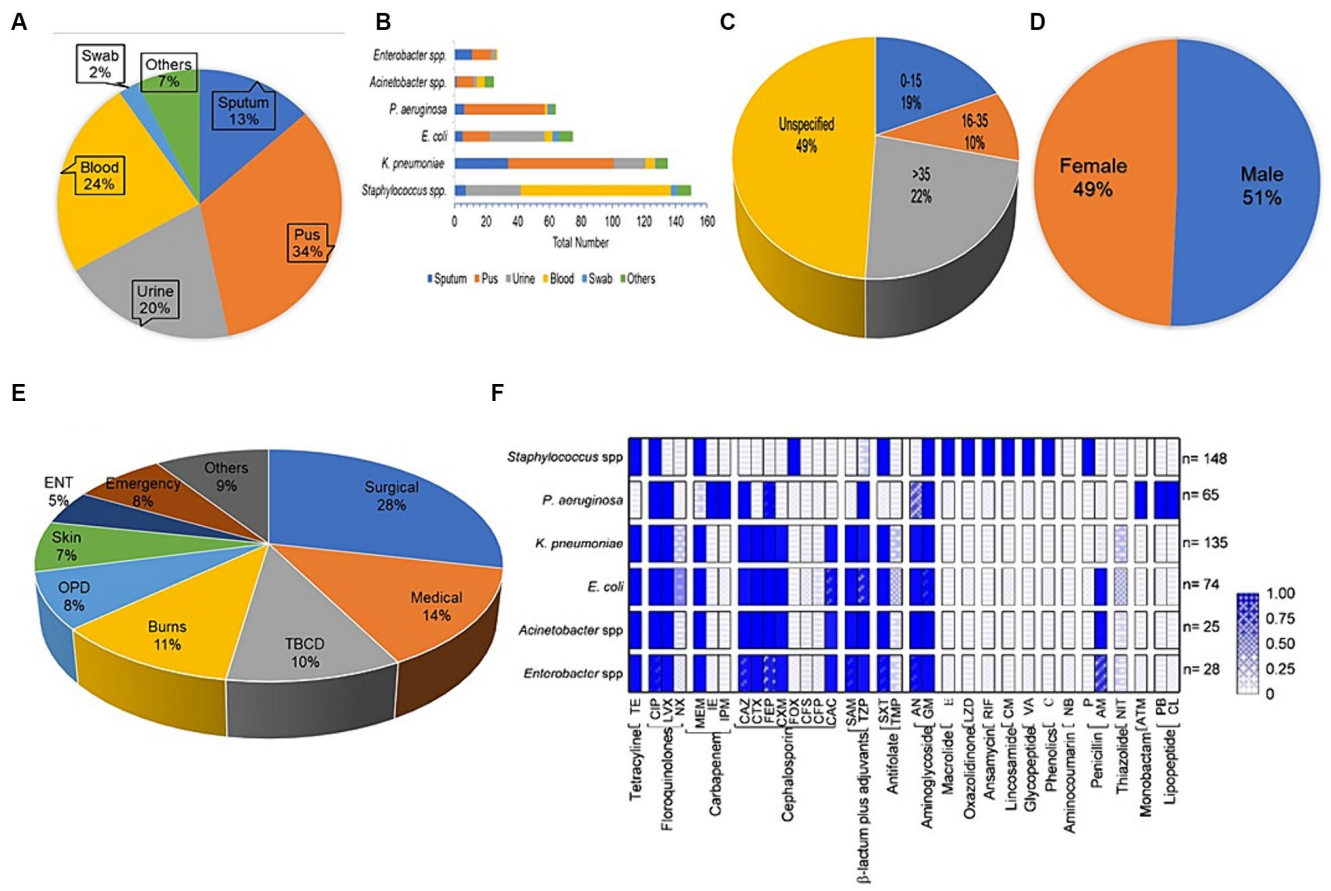
Datasets for antibiotic susceptibility tests. **(A)** Distribution of the specimens gathered for this investigation, **(B)** the source of the pathogenic bacterial strains, **(C)** Age-wise distribution of bacterial isolates, **(D)** Sample distributions by gender, **(E)** Prevalence of bacterial isolates in various wards of the hospital, **(F)** the frequency of testing for different antibiotics (grouped by antibiotic class) against bacterial strains.

475 clinical specimens in the present study exhibited bacterial growth; the majority (34%) of the 475 isolates originated from pus, followed by blood (24%), urine (20%), and sputum (13%), but other isolation sources were common for some pathogens (Figure 1A). The results of the chi-square test revealed that bacterial abundance in specimens was significantly different (*p* value <0.05 Pearson chi square test). The relative abundance of *Staphylococcus* spp. (148; 31%) were isolated most commonly from the collected specimen samples, i.e., 95 of the 148 isolates were observed to be prevalent in blood. *Klebsiella pneumoniae* (135; 28%) was the second most common isolate; that was most prevalent in pus and sputum specimens, 67 and 34, respectively. *Escherichia coli* (74; 15%) was prevalent in urine specimens 35. *Pseudomonas aeruginosa* (65; 14%) pus specimens 51 were most prevalent, followed by *Enterobacter* spp. (28; 6%), equally distributed in pus and sputum, and the majority of *Acinetobacter* spp. (25; 5%) from pus (Figure 1B). The most commonly tested (frequency > 0.9) antibiotics against all the studied bacterial pathogens were aminoglycosides, carbapenems, cephalosporins, and fluoroquinolones. Other classes of antibiotics were often tested for diverse infections; antifolates and tetracyclines were routinely tested against *Staphylococcus* spp., *K*. *pneumoniae*, *E*. *coli*, *Enterobacter* spp., *Acinetobacter* spp. except *P*. *aeruginosa*. Monobactam and lipopeptide-class antibiotics were tested only against *P*. *aeruginosa*, whereas macrolide, anisomycin, lincosamide, glycopeptide, etc. were tested against *Staphylococcus* spp. (Figure 1F). Patients were classified into three age groups, *viz*., 0–15 years (19%), 16–35 years (10%), >35 years (23%), and unknown age (49%) (Figure 1C; Table 1).

**Table 1:** Bacterial pathogens distribution with age group.

Age group	*Enterobacter* spp.	*P*. *aeruginosa*	*Staphylococcus* spp.	*E*. *coli*	*K*. *pneumoniae*	*Acinetobacter* spp.
0–15	2	8	56	4	12	7
16–35	3	3	13	4	21	2
>35	8	17	24	28	25	5
Unspecified	15	37	55	38	77	11
Total	28	65	148	74	135	25

### 3.2 Antibiotic resistance index in bacterial pathogens

The penicillin resistance index (R_I_) of *Staphylococcus* spp. was proportionately 1 (100%)and against ciprofloxacin, cefoxitin, and erythromycin was >0.8. Chloramphenicol was the most effective antibiotic against *Staphylococcus* spp. (Supplementary Figure S1A). *Pseudomonas aeruginosa* exhibited a 0.6 R_I_ to imipenem, ceftazidime, cefepime, and piperacillin-tazobactam. Imipenem-EDTA, and amikacin were most effective against *P*. *aeruginosa* (Supplementary Figure S1B). *Acinetobacter* spp. exhibited *R*_I_ > 0.8 against ciprofloxacin, gentamycin, ceftazidime, cefotaxime, piperacillin-tazobactam, ampicillin-sulbactam, and trimethoprim-sulfamethoxazole; however, meropenem resistance was <0.4 (Supplementary Figure S1C). *Klebsiella pneumoniae* exhibited high resistance against all antibiotics tested except tetracyclines (Supplementary Figure S1D). *Enterobacter* spp. exhibited a *R*_I_ of 0.5 to tetracyclines and amikacin but were more resistant (>0.6) to the other antibiotics (Supplementary Figure S1E). *Escherichia coli* was more sensitive to amikacin (<0.3), and highly resistant to other antibiotics (>0.7) (Supplementary Figure S1F). Similar report by Gupta et al. (2014) stated that uropathogenic *E*. *coli*, *Enterococcus faecalis*, *K*. *pneumoniae*, *Staphylococcus aureus*, *P*. *aeruginosa*, and *Proteus mirabilis* revealed ampicillin resistance up to 94–100%. Uropathogenic *E*. *coli* strains prevalent in Rajasthan, India, exhibited 95% resistance to nalidixic acid and 80% resistance to ampicillin and amoxiclav antibiotics (Sood and Gupta, 2012).

### 3.3 Does the antibiotic resistance pattern of bacterial pathogens vary with isolation sources?

*Klebsiella pneumoniae* spp. isolated from patients of medical and surgical wards exhibited a strong antibiotic-resistance correlation 0.85 (Table 2). Similarly, *K*. *pneumoniae* strains obtained from patients in medical ward and intensive care unit (ICU) exhibited antibiotic-resistance correlation 0.79. *Escherichia coli* isolates showed strong antibiotic-resistance correlation 0.96, 0.87, and 0.82 between the out-patient department (OPD) and medical ward, OPD and surgical ward, and surgical-medical wards, respectively. *Pseudomonas aeruginosa* isolated from skin-burns, skin-surgical, and burns-surgical wards, showed substantial antibiotic-resistance correlation of 0.97, 0.93, and 0.91, respectively. *Staphylococcus* spp. exhibited a strong antibiotic-resistance correlation 0.98 between medical and surgical wards, but a moderate antibiotic-resistance correlation 0.59 between Emergency and tuberculosis chest diseases (TBCD) wards. These findings strongly suggest that the antibiotic resistance patterns of the above-mentioned isolates were similar with respect to isolation sources. However, differences were also observed in the antibiotic resistance patterns of bacterial pathogens isolated from the patients of other wards; e.g., *K*. *pneumoniae* isolates revealed a weak antibiotic-resistance correlation 0.29 between burns and ICU wards. Similarly, *E*. *coli* exhibited an antibiotic-resistance correlation 0.30 between the ICU and medical ward and *P*. *aeruginosa* isolates showed antibiotic-resistance correlation 0.14 and 0.22 between the TBCD-ear nose throat (ENT) and burns-ENT wards, respectively (Kelch and Lee, 1978). There is ample published literature describing the antibiotic sensitivity phenotype of non-clinical *P*. *aeruginosa*, *E*. *coli*, *Enterobacter* spp., *K*. *pneumoniae*, and *Staphylococcus* spp. (Supplementary Table S2).

**Table 2:** Antibiotic resistance correlation matrix for bacterial pathogens isolated from various wards of hospital.

*K*. *pneumoniae*	Medical	Surgical	ICU	TBCD	Burns				
Medical	1								
Surgical	0.85	1							
ICU	0.79	0.48	1						
TBCD	0.64	0.75	0.48	1					
Burns	0.65	0.73	0.29	0.70	1				
*E*. *coli*	Medical	Surgical	ICU	TBCD	OPD				
Medical	1								
Surgical	0.82	1							
ICU	0.30	0.46	1						
TBCD	0.77	0.57	0.14	1					
OPD	0.96	0.87	0.42	0.75	1				
*P*. *aeruginosa*	ENT	Surgical	TBCD	Burns	Emergency	Skin			
ENT	1								
Surgical	0.48	1							
TBCD	0.14	0.88	1						
Burns	0.22	0.91	0.87	1					
Emergency	0.76	0.86	0.65	0.75	1				
Skin	0.29	0.93	0.86	0.97	0.79	1			
*Staphylococcus* spp.	OPD	Children	Surgical	ICU	TBCD	Medical	Emergency	Skin	ENT
OPD	1								
Children	0.86	1							
Surgical	0.96	0.91	1						
ICU	0.89	0.91	0.91	1					
TBCD	0.86	0.77	0.79	0.78	1				
Medical	0.96	0.91	0.98	0.93	0.82	1			
Emergency	0.82	0.78	0.86	0.92	0.59	0.86	1		
Skin	0.95	0.87	0.94	0.84	0.82	0.95	0.74	1	
ENT	0.90	0.63	0.83	0.71	0.85	0.82	0.61	0.8434	1

ICU, Intensive care unit; TBCD, Tuberculosis chest disease; OPD, Out-patient department; ENT, Ear nose throat.

### 3.4 Changing antibiotic sensitivity phenotypes of pathogens

**Figure 2:**
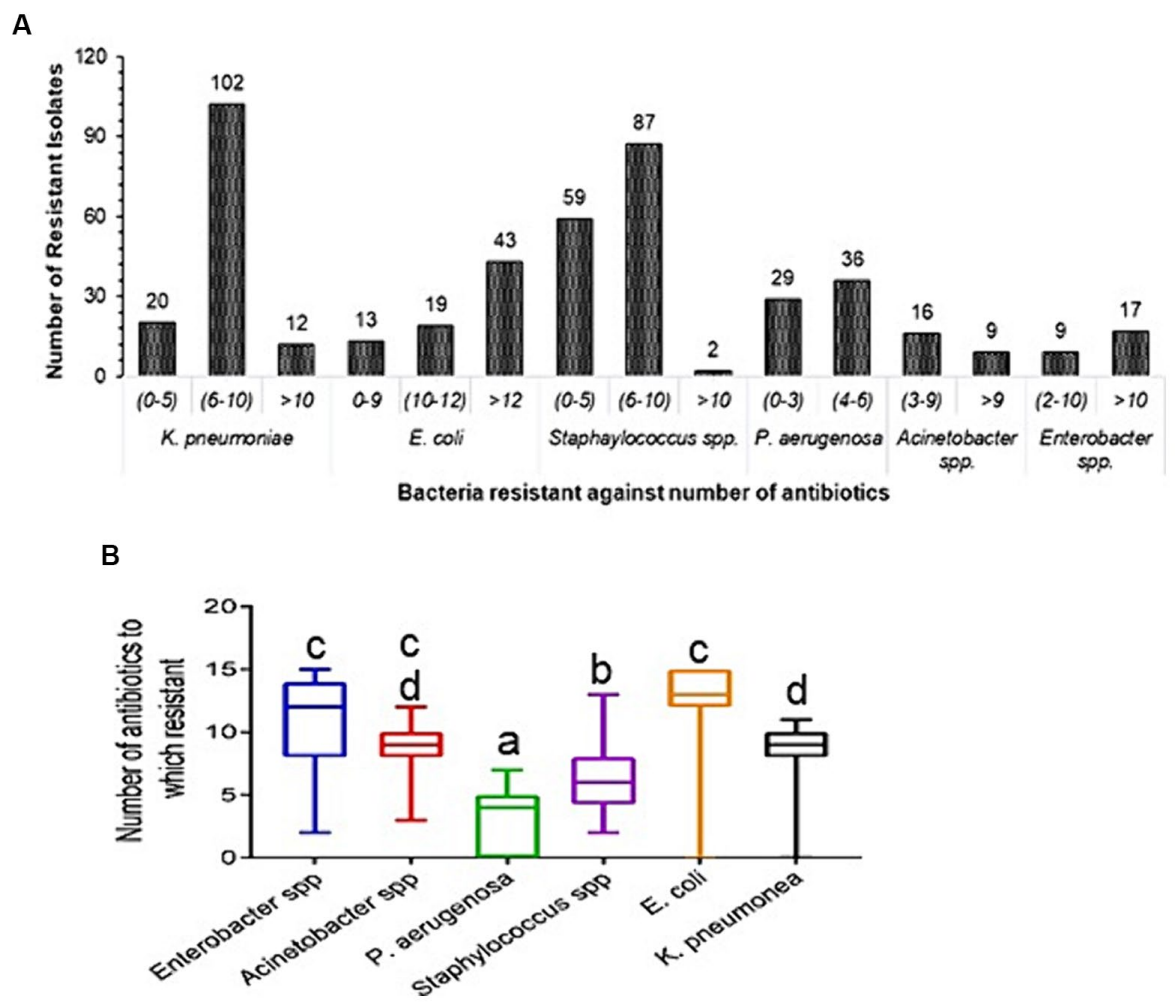
Prevalence of antibiotic resistance in clinical strains. **(A)** Diversity of antibiotic resistance in clinical isolates. Each bar represents the number of isolates that were resistant to antibiotics. **(B)** Total number of drugs for which resistance was found. It is plotted using box plots; the standard deviation is indicated by the error bar on both sides, and the middle line displays the mean value of the number of antibiotics resistant to each type of bacteria. Tukey *post-hoc* test was conducted for comparisons between the mean values of number of resistant antibiotics, *p* < 0.05 was considered statistically significant. Diference alphabets indicates statistical significant.

*Klebsiella pneumoniae* (102) and *Staphylococcus* (87) isolates were resistant to 6–10 tested antibiotics; *E*. *coli* (43) strains were resistant to >12 antibiotics; and *P*. *aeruginosa* (36) isolates were resistant to 4–6 antibiotics (Figure 2A). *Enterobacter* spp. and *E*. *coli* show 12 and 13 mean values for the number of resistant antibiotics, respectively. Whereas *P*. *aeruginosa* and *Staphylococcus* spp. only have four and six mean values for the number of antibiotic resistant. The mean values of number of resistant antibiotics (9) were not significant for *K*. *pneumoniae* and *Acinetobacter* spp. (*p* = 0.80). The comparison of *Enterobacter* spp. with other bacteria was significant (*p* < 0.05), except for *E*. *coli* and *Acinetobacter* spp. (*p* = 0.167) (Figure 2B). In the current study, we classified pathogens into three selection groups *viz*. directional selection, disruptive selection, and stabilizing selection. *Klebsiella pneumoniae* isolates were observed to undergo directional selection toward resistant phenotypes in gaining resistance to antibiotics CIP, LVX, FEP, TZP, and CXM; while it showed evolutionary disruptive selection toward antibiotics SXT, CAZ, CTX, GM, AN, TE, SAM, and CAC; and appeared to adopt stabilizing selection to antibiotic MEM in (Supplementary Figure S2A). *Escherichia coli* showed directional selection toward resistant phenotypes to antibiotics CIP, LVX, CAZ, FEP, TZP, CTX, and CXM, disruptive selection to antibiotics SXT, GM, TE, SAM, MEM, and CAC, and stabilizing selection to antibiotic AN (Supplementary Figure S2B). *Pseudomonas aeruginosa* isolates exhibited directional selection toward sensitive phenotypes to antibiotics ATM and IE, while toward resistant phenotype to antibiotic CIP, and disruptive selection to antibiotics GM, AN, CAZ, FEP, TZP, and IPM (Supplementary Figure S2C). *Staphylococcus* spp. were observed to adopt directional selection toward resistant phenotype to antibiotics CIP and E, and disruptive selection to antibiotics SXT, GM, TE, CM, C, and RIF (Supplementary Figure S2D). The selection patterns for *Enterobacter* and *Acinetobacter* spp. isolates are drawn in (Supplementary Figures S2E,F).

### 3.5 Comparison of antibiotic susceptibility between clinical and non-clinical isolates

**Figure 3:**
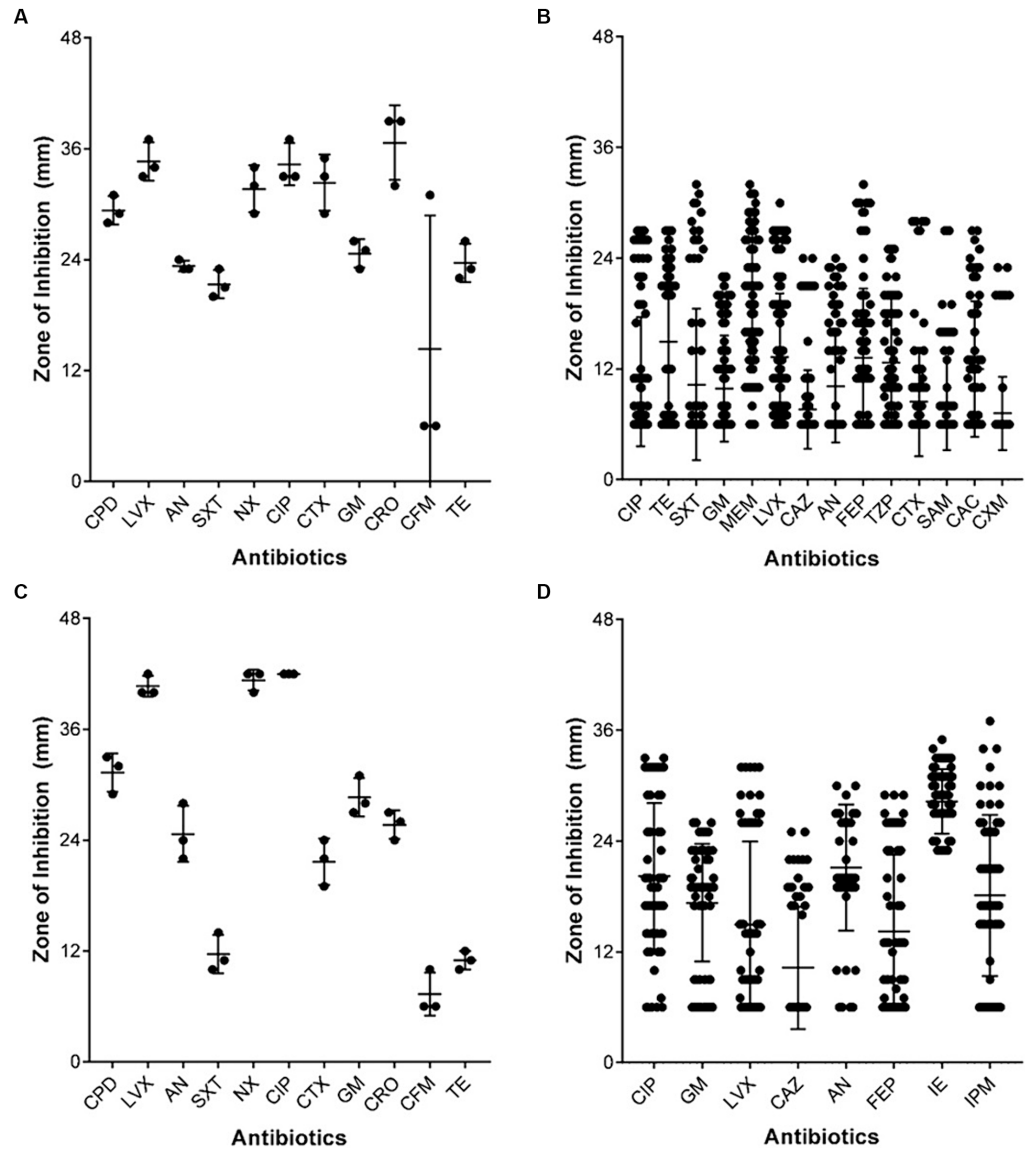
Comparison of the size of antibiotic susceptibility zone of Gram-negative bacteria. **(A)** *Klebsiella pneumoniae* isolated from *Spodoptera frugiperda* caterpillar carcasses, **(B)** *Klebsiella pneumoniae* isolated from clinical samples, **(C)** *Pseudomonas aeruginosa* isolated from *Spodoptera frugiperda* caterpillar carcasses, and **(D)** *Pseudomonas aeruginosa* isolated from clinical samples. Box plots were used for the comparison; each black dot represents an isolated bacterial strain, the central line shows the mean value of the zone of inhibition, and the error bar on both sides reflects the standard deviation.

Non-clinical *P*. *aeruginosa* isolated from caterpillar carcasses was more sensitive compared to clinical isolates against fluoroquinolones LVX, CIP, and NX, third generation cephalosporins cefpodoxime (CPD), ceftriaxone (CRO), and CTX, amikacin and gentamicin. The zone of inhibition of non-clinical *P*. *aeruginosa* was 1.1–1.5 times bigger than those of clinical *P*. *aeruginosa* against CIP and LVX, indicating higher resistance of clinical than non-clinical *P*. *aeruginosa* (Figures 3C,D). Similarly, clinical *K*. *pneumoniae* isolates tended to be resistant to LVX and CIP and second and third generation cephalosporins, while most being resistant also to fourth generation cephalosporins. Non-clinical *K*. *pneumoniae* strains were sensitive to CIP, LVX, and NX, third generation cephalosporins CPD, CTX, and CRO, with the exception of cefixime (CFM), and to AN and GM. More than 60% of clinical *K*. *pneumoniae* strains were resistant to the antibiotics AN and GM (Figures 3A,B).

## 4 Discussion

This study describes the prevalence of antibiotic resistance in bacterial pathogens isolated from clinical samples at PDU Hospital Rajkot, Gujarat. We observed that 52% patients were infected with pathogenic bacteria and the number of male and female patients carrying bacterial pathogens was nearly the same (Figure 1D). Our analysis of the antibiotic sensitivity of pathogenic bacterial strains revealed that around half of the bacterial strains (237; 49.89%) were from the patients of the surgical wards, medical wards and ICU of the hospital, and the remaining pathogenic bacterial strains (238; 50.1%) were isolated from more than 10 other wards of the hospital (Figure 1E). The development and spread of antibiotic resistance among bacterial pathogens has been a continuously growing global problem. Pathogens were categorized as methicillin-resistant, and EICR *Staphylococcus* spp., *K*. *pneumoniae*, *E*. *coli*, and *Enterobacter* spp. were categorized into ESBL, *P*. *aeruginosa* in MBL category based on the latest CLSI guidelines (CLSI, 2022).

Our data analysis on antibiotic resistance and susceptible antimicrobial patterns in some instances contradicts while in other instances supports the results of earlier national and international research. In this study, 8.8 and 20% ESBL strains of *K*. *pneumoniae* and *E*. *coli* were, respectively, isolated, which is similar to the figure reported (Sood and Gupta, 2012). While, Mohapatra et al., reported that 44.8% ESBL uropathogens infection were observed in the community in India (Mohapatra et al., 2022). In China, >50% bacterial pathogens isolated during 2000–2009, were MRSA, ESBL Enterobacteriaceae, and carbapenem-resistant *P*. *aeruginosa* (Xiao et al., 2011), which is considerably high than values we report. In the current study, *P*. *aeruginosa*, *K*. *pneumoniae*, *E*. *coli*, *Enterobacter* spp., *Acinetobacter* spp., and *Staphylococcus* spp. were observed to be highly resistant to the third and fourth generation cephalosporins (Table 3). These pathogens were found in the skin, surgical, emergency, and pediatric wards, as well as the surgical, medical, and ICU wards. 70–90% Enterobacterales were resistant to fluoroquinolones, and the six bacterial pathogens studied (70–100%) were resistant to third generation cephalosporin, which is marginally higher than the recent reports (Diallo et al., 2020; Mannathoko et al., 2022; Murray et al., 2022). Meropenem, tetracycline, gentamycin, and amikacin effectively controlled enteric pathogens. Chloramphenicol, linezolid, vancomycin, tetracycline, clindamycin, and rifamycin were effective against *Staphylococcus* spp., but penicillin, ciprofloxacin, cefoxitin, and erythromycin were poor. 21.6% *Staphylococcus* spp. were methicillin-resistant and 7.4% were EICR. MRSA and EICR *Staphylococcus* strains have been reported to be isolated, albeit at a higher frequency (Gandra et al., 2016; Murray et al., 2022). In our study, Imipenem, amikacin, imipenem-EDTA, and gentamycin were most effective against *P*. *aeruginosa*. *P*. *aeruginosa* with MBL activity (14; 2.9%) showed similarity with earlier reports (Gandra et al., 2016; Lob et al., 2022). Levofloxacin and amikacin are effective against *Acinetobacter* spp. and (32%) resistant to MEM, which is comparatively lower than the previously reported 87.2% in India and 88% in South Korea (Gandra et al., 2016; Nordmann and Poirel, 2019; Murray et al., 2022; Lee et al., 2023). Multidrug resistant *E*. *coli*, *K*. *pneumoniae*, and *Acinetobacter baumannii*, methicillin-resistant *Staphylococcus* spp. reportedly increase mortality rates two to three times, in hospitalized patients in India (Gandra et al., 2019). In this study, *K*. *pneumoniae* and *E*. *coli* were resistant to more than 6–12 antibiotics, while *P*. *aeruginosa* was resistant to 6–10 antibiotics, somewhat lower than a recent report (Bassetti et al., 2019). *Staphylococcus* spp. resistant to 4–6 antibiotics (Figure 2B) comparable to earlier report (Kumar et al., 2017). Unscrupulous and haphazard administration of antibiotics is largely, if not entirely, responsible for the evolution of antibiotic resistance among various isolates (Beckley and Wright, 2021). Comparison of antibiotic susceptibility between clinical and non-clinical isolates can be important in the evaluation of the development and evolution of antibiotic resistance among clinical and non-clinical strains of human pathogens. Since clinical bacterial strains are regularly exposed to antibiotics and therefore exhibit directional selection toward resistance traits, they are more resistant than non-clinical strains. Non-clinical *P*. *aeruginosa* and *K*. *pneumoniae* were highly sensitive to tested antibiotics (Figures 3A,C). The antibiotic resistant rate in non-clinical *P*. *aeruginosa*, *K*. *pneumoniae*, *E*. *coli*, *Enterobacter* spp., and *Staphylococcus* spp. is comparatively lower than the clinical strains (Supplementary Table S2) and similar results was reported on antimicrobial resistance in animals (Van Boeckel et al., 2019). Environmental pressure is responsible for natural selection in every organism, which helps enhance the fitness of organisms. Three types of natural selection were observed (1) directional selection, (2) stabilizing selection, and (3) disruptive selection (Darwin, 1859). Presence of antibiotics in the surrounding environment is one of the factors responsible for natural selection pressure in bacteria (Baquero, 2001; Blazquez et al., 2002). Our analysis in the present study of enteric pathogens showed directional selection toward resistant phenotypes against TZP, FEP, and CXM, which are therefore less effective. All six bacterial pathogens in present study showed directional selection toward resistant phenotypes against CIP, and its effectiveness was very poor. Inappropriate use of antibiotics, and inappropriate timing of use (pre/post-operative antimicrobial prophylaxis) have been described as some of the factors responsible for the development of antibiotic resistance in bacteria (Goldmann, 1999; Thu et al., 2012; Lim et al., 2015). Such directional selection in *P*. *aeruginosa* (Barbosa et al., 2017) and an increased selection coefficient in response to a high concentration of cefotaxime in *E*. *coli* have been reported earlier for directional selection of antibiotic resistant phenotypes in bacteria (Negri et al., 2000). Such types of directional selection of antibiotic resistant phenotypes in bacteria make it more challenging to control these kinds of outbreaks. A comparison of the present research findings with results from earlier research can offer some validation of the findings of this present study and also offer methodological variations in their approaches. However, our findings contribute to regional and worldwide databases on the susceptibility and effectiveness of antibiotics against clinical isolates in this geographical area. This will help the clinicians of various hospitals in this region formulate empirical antimicrobial therapy and proper infection control measures. This study can be useful in studying the patterns of rising resistance among clinical bacterial isolates in this particular region of the country.

**Table 3:** Antibiotic resistance in clinical bacterial pathogens isolated from various wards of PDU Hospital, Rajkot.

Isolates	Hospital wards (*n* = Isolate)	Antibiotics resistance (%)														
		CIP	LVX	SXT	GM	AN	TE	CAZ	FEP	TZP	CTX	SAM	MEM	CAC	CXM	AMP
*K*. *pneumoniae*	Medical (21)	62	62	48	48	52	43	71	67	67	71	67	38	62	71	
	Surgical (37)	92	94	85	85	88	79	100	85	94	100	91	59	97	100	
	ICU (10)	90	50	30	50	70	10	100	100	100	100	100	70	100	100	
	TBCD (20)	85	25	90	80	55	35	100	57	80	100	90	5	80	100	
	Burns (30)	93	87	93	90	83	43	97	90	87	93	87	17	50	93	
*E*. *coli*	Medical (21)	100	95	86	86	76	95	100	95	95	100	95	67	95	100	
	Surgical (16)	100	100	94	100	69	81	100	100	100	100	100	69	100	100	
	ICU (5)	80	80	100	80	60	80	100	80	80	80	80	80	100	80	
	TBCD (4)	100	75	75	75	75	75	100	75	75	100	75	50	75	100	
	OPD (13)	100	77	77	54	31	69	100	92	92	100	92	8	92	100	
*Enterobacter* spp.	Surgical (11)	100	100	45	91	36	82	100	100	91	100	91		100	100	100
	TBCD (6)	50	50	33	33	33	33	83	50	33	100	33		50	100	100
	Burns (3)	100	33	100	100	100	67	100	100	100	100	100		100	100	100
		CIP	LVX	SXT	GM	AN	TE	CAZ	FEP	TZP	CTX	SAM	MEM			
*Acinetobacter* spp.	Surgical (9)	100	0	100	100	0	100	100	33	100	100	100	0			
	TBCD (3)	67	0	67	67	33	67	100	33	67	100	100	33			
	ICU (4)	100	100	100	100	100	50	100	75	100	100	100	75			
	Emergency (3)	100	0	100	100	100	0	100	100	100	100	100	100			
		CIP	GM	AN	CAZ	FEP	TZP	IE	IPM	ATM						
*P*. *aeruginosa*	ENT (13)	46	15	0	31	31	31	0	0	31						
	Surgical (17)	53	41	29	100	88	82	0	71	29						
	ICU (4)	0	0	0	0	0	0	0	0	0						
	TBCD (5)	0	0	0	20	20	20	0	20	0						
	Burns (10)	30	20	50	90	70	80	0	60	10						
	Emergency (8)	63	38	33	100	88	100	0	38	63						
	Skin (6)	50	33	67	100	100	100	0	100	0						
		CIP	E	SXT	GM	LZD	TE	P	FOX	CM	MEM	VA	C	RIF		
*Staphylococcus* spp.	OPD (12)	92	92	18	36	18	0	100	73	27	45	9	0	9		
	Children (18)	94	67	50	83	0	11	100	100	50	72	11	0	22		
	Surgical (30)	100	87	13	57	13	10	100	93	33	70	23	3	20		
	ICU (39)	74	87	44	46	13	31	100	97	59	82	15	13	31		
	TBCD (4)	100	75	50	25	25	0	100	75	75	25	25	0	25		
	Medical (11)	91	91	18	64	18	18	100	91	55	64	27	9	27		
	Emergency (4)	50	100	25	50	25	25	100	100	50	100	25	25	25		
	Skin (20)	100	100	40	70	25	30	100	70	50	60	45	10	40		
	ENT (4)	100	75	0	0	25	0	100	50	25	25	25	0	25		

ICU, Intensive care unit; TBCD, Tuberculosis chest disease; OPD, Out-patient department; ENT, Ear nose throat.

## 5 Conclusion

The study findings offer a valuable resource for cross-national and within-country comparisons of the antimicrobial resistance patterns among PEEKSA isolates in Gujarat, India. Cephalosporin and fluoroquinolone antibiotics show poor activity in controlling bacterial pathogens. Tetracyclines and aminoglycosides effectively control *Escherichia coli*, *Enterobacter* spp., *Klebsiella pneumoniae*, and *Acinetobacter* spp. Chloramphenicol, linezolid, vancomycin, tetracycline, and rifamycin are the most effective antibiotics in descending order to control *Staphylococcus* spp. Imipenem-EDTA was the most effective treatment for *Pseudomonas aeruginosa*, followed by gentamycin, amikacin, and imipenem. Bacterial pathogens isolated from the surgical ward were comparatively more antibiotic resistant than those isolated from other wards. The widespread administration of antimicrobial agents and antibiotics in surgical wards appears to be the apparent reason. Non-clinical *P*. *aeruginosa* and *K*. *pneumoniae* were more sensitive to antibiotics than the clinical isolates. Unscrupulous and haphazard use of antibiotics to treat bacterial diseases increases the selection pressure toward resistance phenotypes in bacteria, narrowing the scope of reversing the directional shift from resistant to sensitive phenotypes. Comprehending the genetic makeup (resistance gene or plasmid) in bacteria exhibiting higher resistance rates could facilitate the understanding of mechanisms, and that will lead to the development of novel antibiotics with innovative mechanisms or more effective therapeutic approaches. Our findings serve as baseline datasets that can be used to link and implicate antibiotic stewardship programs in hospitals in this region of the country.

## Data availability statement

The original contributions presented in the study are included in the article/Supplementary material, further inquiries can be directed to the corresponding author.

## Ethics statement

Data collection and sample processing were done under the SOP guidelines and regulations of the PDU Medical College ethical panel. The Ethics Committee of PDU Medical College waived the requirement for informed consent.

## Author contributions

VH: Conceptualization, Data curation, Formal Analysis, Investigation, Methodology, Software, Writing – original draft, Writing – review & editing. BP: Data curation, Formal Analysis, Software, Writing – review & editing. RK: Supervision, Writing – review & editing. AB: Data curation, Methodology, Validation, Writing – review & editing. GK: Conceptualization, Formal Analysis, Resources, Writing – review & editing. BV: Conceptualization, Methodology, Supervision, Validation, Visualization, Writing – review & editing.

## References

[ref1] Akiba M. Senba H. Otagiri H. Prabhasankar V. P. Taniyasu S. Yamashita N.. (2015). Impact of wastewater from different sources on the prevalence of antimicrobial-resistant *Escherichia coli* in sewage treatment plants in South India. Ecotoxicol. Environ. Saf. 115, 203–208. doi: 10.1016/j.ecoenv.2015.02.018, PMID: 25704279

[ref2] Bai A. D. Lo C. K. Komorowski A. S. Suresh M. Guo K. Garg A.. (2022). *Staphylococcus aureus* bacteremia mortality across country income groups: a secondary analysis of a systematic review. Int. J. Infect. Dis. 122, 405–411. doi: 10.1016/j.ijid.2022.06.026, PMID: 35728748

[ref3] Baquero F. (2001). Low-level antibacterial resistance: a gateway to clinical resistance. Drug Resist. Updat. 4, 93–105. doi: 10.1054/drup.2001.0196, PMID: 11512526

[ref4] Baquero F. Martinez J. L. Lanza F. V. Rodríguez-Beltrán J. Galán J. C. San Millán A.. (2021). Evolutionary pathways and trajectories in antibiotic resistance. Clin. Microbiol. Rev. 34:e0005019. doi: 10.1128/CMR.00050-19, PMID: 34190572 PMC8404696

[ref5] Barbosa C. Trebosc V. Kemmer C. Rosenstiel P. Beardmore R. Schulenburg H.. (2017). Alternative evolutionary paths to bacterial antibiotic resistance cause distinct collateral effects. Mol. Biol. Evol. 34, 2229–2244. doi: 10.1093/molbev/msx158, PMID: 28541480 PMC5850482

[ref6] Bassetti M. Castaldo N. Cattelan A. Mussini C. Righi E. Tascini C.. (2019). Ceftolozane/tazobactam for the treatment of serious *Pseudomonas aeruginosa* infections: a multicentre nationwide clinical experience. Int. J. Antimicrob. Agents 53, 408–415. doi: 10.1016/j.ijantimicag.2018.11.001, PMID: 30415002

[ref7] Beckley A. M. Wright E. S. (2021). Identification of antibiotic pairs that evade concurrent resistance via a retrospective analysis of antimicrobial susceptibility test results. Lancet Microb. 2, e545–e554. doi: 10.1016/S2666-5247(21)00118-X, PMID: 34632433 PMC8496867

[ref8] Blazquez J. Oliver A. Gomez-Gomez J. M. (2002). Mutation and evolution of antibiotic resistance: antibiotics as promoters of antibiotic resistance? Curr. Drug Targets 3, 345–349. doi: 10.2174/1389450023347579 12102604

[ref9] Britto C. D. John J. Verghese V. P. Pollard A. J. (2019). A systematic review of antimicrobial resistance of typhoidal *Salmonella* in India. Indian J. Med. Res. 149:151. doi: 10.4103/ijmr.IJMR_830_18 31219079 PMC6563740

[ref10] Browne A. J. Chipeta M. G. Haines-Woodhouse G. Kumaran E. P. Hamadani B. H. K. Zaraa S.. (2021). Global antibiotic consumption and usage in humans, 2000–18: a spatial modelling study. Lancet Planet. Health 5, e893–e904. doi: 10.1016/S2542-5196(21)00280-1, PMID: 34774223 PMC8654683

[ref11] CDC (2019). Antibiotic Resistance Threats in the United States. Atlanta, GA: U.S. Department of Health and Human Services, CDC.

[ref12] Cheesbrough M. (2005). District Laboratory Practice in Tropical Countries, Part 2. New York: Cambridge University Press

[ref13] CLSI (2018). Methods for dilution antimicrobial susceptibility tests for Bacteria that grow aerobically. CLSI standard M07, 11th Edn. Wayne, PA. Clinical and Laboratory Standard Institite.

[ref14] CLSI (2022). Performance standards for antimicrobial susceptibility testing. 32nd ed. CLSI supplement M100.

[ref15] Collee J. G. Miles R. S. Watt B. (1996). “Tests for the identification of Bacteria” in Practical Medical Microbiology. eds. Collee J. G. Marmion B. P. Fraser A. G. Mackie S. A.. McCartney Practical Medical Microbiology, 14th ed (New York: Churchill Livingstone), 131–150.

[ref16] Darwin C. (1859). On the Origins of Species by Means of Natural Selection 247. London: Murray, 1859

[ref17] Diallo O. O. Baron S. A. Dubourg G. Chaudet H. Halfon P. Camiade S.. (2020). Major discrepancy between factual antibiotic resistance and consumption in south of France: analysis of 539,037 bacterial strains. Sci. Rep. 10:18262. doi: 10.1038/s41598-020-75158-7, PMID: 33106494 PMC7588456

[ref18] Gandra S. Joshi J. Trett A. Lamkang A. S. Laxminarayan R. (2017). Scoping report on antimicrobial resistance in India. Center for Disease Dynamics, Economics & Policy: Washington, DC, USA, 2017, 1–146.

[ref19] Gandra S. Mojica N. Klein E. Y. Ashok A. Nerurkar V. Kumari M.. (2016). Trends in antibiotic resistance among major bacterial pathogens isolated from blood cultures tested at a large private laboratory network in India, 2008–2014. Int. J. Infect. Dis. 50, 75–82. doi: 10.1016/j.ijid.2016.08.002, PMID: 27522002 PMC5063511

[ref20] Gandra S. Tseng K. K. Arora A. Bhowmik B. Robinson M. L. Panigrahi B.. (2019). The mortality burden of multidrug-resistant pathogens in India: a retrospective, observational study. Clin. Infect. Dis. 69, 563–570. doi: 10.1093/cid/ciy955, PMID: 30407501 PMC6669283

[ref21] Goldmann D. A. (1999). The epidemiology of antimicrobial resistance. Ecosyst. Health 5, 158–163. doi: 10.1046/j.1526-0992.1999.09925.x

[ref22] Gupta S. Kapur S. Padmavathi D. V. (2014). Comparative prevalence of antimicrobial resistance in community-acquired urinary tract infection cases from representative states of northern and southern India. J. Clin. Diagn. Res. 8:DC09. doi: 10.7860/JCDR/2014/9349.4889 PMC4225884 25386432

[ref23] Ikuta K. S. Swetschinski L. R. Aguilar G. R. Sharara F. Mestrovic T. Gray A. P.. (2022). Global mortality associated with 33 bacterial pathogens in 2019: a systematic analysis for the global burden of disease study 2019. Lancet 400, 2221–2248. doi: 10.1016/S0140-6736(22)02185-7, PMID: 36423648 PMC9763654

[ref24] Kaur A. Gandra S. Gupta P. Mehta Y. Laxminarayan R. Sengupta S. (2017). Clinical outcome of dual colistin-and carbapenem-resistant *Klebsiella pneumoniae* bloodstream infections: a single-center retrospective study of 75 cases in India. Am. J. Infect. Control 45, 1289–1291. doi: 10.1016/j.ajic.2017.06.028, PMID: 28807425

[ref25] Kelch W. J. Lee J. S. (1978). Antibiotic resistance patterns of gram-negative bacteria isolated from environmental sources. Appl. Environ. Microbiol. 36, 450–456. doi: 10.1128/aem.36.3.450-456.1978, PMID: 727777 PMC243068

[ref26] Klein E. Y. Tseng K. K. Pant S. Laxminarayan R. (2019). Tracking global trends in the effectiveness of antibiotic therapy using the drug resistance index. BMJ Glob. Health 4:e001315. doi: 10.1136/bmjgh-2018-001315, PMID: 31139449 PMC6509601

[ref27] Kumar P. Bag S. Ghosh T. S. Dey P. Dayal M. Saha B.. (2017). Molecular insights into antimicrobial resistance traits of multidrug resistant enteric pathogens isolated from India. Sci. Rep. 7:14468. doi: 10.1038/s41598-017-14791-1, PMID: 29089611 PMC5663842

[ref28] Larsson D. G. Flach C. F. (2022). Antibiotic resistance in the environment. Nat. Rev. Microbiol. 20, 257–269. doi: 10.1038/s41579-021-00649-x, PMID: 34737424 PMC8567979

[ref29] Lee Y. L. Ko W. C. Hsueh P. R. (2023). Geographic patterns of *Acinetobacter baumannii* and carbapenem resistance in the Asia-Pacific region: results from the antimicrobial testing leadership and surveillance (ATLAS) program, 2012-2019. Int. J. Infect. Dis. 127, 48–55. doi: 10.1016/j.ijid.2022.12.010, PMID: 36516915

[ref30] Lim M. K. Lai P. S. M. Ponnampalavanar S. S. L. S. Omar S. F. S. Taib N. A. Yusof M. Y.. (2015). Antibiotics in surgical wards: use or misuse? A newly industrialized country’s perspective. J. Infect. Develop. Countries 9, 1264–1271. doi: 10.3855/jidc.6731 26623636

[ref31] Lob S. H. Estabrook M. A. DeRyke C. A. Alekseeva I. Siddiqui F. Young K.. (2022). Activity of ceftolozane/tazobactam against clinical isolates of *Pseudomonas aeruginosa* from patients in the Middle East and Africa–study for monitoring antimicrobial resistance trends (SMART) 2017-2020. Int. J. Infect. Dis. 125, 250–257. doi: 10.1016/j.ijid.2022.10.014 36244599

[ref32] Mannathoko N. Mosepele M. Gross R. Smith R. M. Alby K. Glaser L.. (2022). Colonization with extended-spectrum cephalosporin-resistant Enterobacterales (ESCrE) and carbapenem-resistant Enterobacterales (CRE) in healthcare and community settings in Botswana: an antibiotic resistance in communities and hospitals (ARCH) study. Int. J. Infect. Dis. 122, 313–320. doi: 10.1016/j.ijid.2022.06.004, PMID: 35688308

[ref33] Mohapatra S. Panigrahy R. Tak V. JV S. KC S. Chaudhuri S.. (2022). Prevalence and resistance pattern of uropathogens from community settings of different regions: an experience from India. Access Microbiology 4:000321. doi: 10.1099/acmi.0.000321 35355869 PMC8941965

[ref34] Murray C. J. Ikuta K. S. Sharara F. Swetschinski L. Aguilar G. R. Gray A.. (2022). Global burden of bacterial antimicrobial resistance in 2019: a systematic analysis. Lancet 399, 629–655. doi: 10.1016/S0140-6736(21)02724-0, PMID: 35065702 PMC8841637

[ref35] Negri M. C. Lipsitch M. Blázquez J. Levin B. R. Baquero F. (2000). Concentration-dependent selection of small phenotypic differences in TEM β-lactamase-mediated antibiotic resistance. Antimicrob. Agents Chemother. 44, 2485–2491. doi: 10.1128/aac.44.9.2485-2491.2000, PMID: 10952599 PMC90089

[ref36] Nordmann P. Poirel L. (2019). Epidemiology and diagnostics of carbapenem resistance in gram-negative bacteria. Clin. Infect. Dis. 69, S521–S528. doi: 10.1093/cid/ciz824, PMID: 31724045 PMC6853758

[ref37] Procop G. W. Church D. L. Hall G. S. Janda W. M. (2020). Koneman's Color Atlas and Textbook of Diagnostic Microbiology. Philadelphia: Jones & Bartlett Learning, 253–320.

[ref38] Sood S. Gupta R. (2012). Antibiotic resistance pattern of community acquired uropathogens at a tertiary care hospital in Jaipur, Rajasthan. Ind. J. Commun. Med. 37, 39–44. doi: 10.4103/0970-0218.94023, PMID: 22529539 PMC3326806

[ref39] Thu T. A. Rahman M. Coffin S. Harun-Or-Rashid M. Sakamoto J. Hung N. V. (2012). Antibiotic use in Vietnamese hospitals: a multicenter point-prevalence study. Am. J. Infect. Control 40, 840–844. doi: 10.1016/j.ajic.2011.10.020, PMID: 22341530

[ref40] Van Boeckel T. P. Pires J. Silvester R. Zhao C. Song J. Criscuolo N. G.. (2019). Global trends in antimicrobial resistance in animals in low-and middle-income countries. Science 365:eaaw1944. doi: 10.1126/science.aaw1944, PMID: 31604207

[ref41] Xiao Y. H. Giske C. G. Wei Z. Q. Shen P. Heddini A. Li L. J. (2011). Epidemiology and characteristics of antimicrobial resistance in China. Drug Resist. Updat. 14, 236–250. doi: 10.1016/j.drup.2011.07.001, PMID: 21807550

