# Supplementary material for: A study of antibiotic resistance pattern of clinical bacterial pathogens isolated from patients in a tertiary care hospital
Source: Front Microbiol. 2024 Apr 17;15:1383989. doi: 10.3389/fmicb.2024.1383989 (PMC11061477; doi:10.3389/fmicb.2024.1383989)
Supplement: Supplementary file 1 [file Data_Sheet_1.PDF]

A study of antibiotic resistance pattern of clinical bacterial pathogens isolated from patients in a tertiary care hospital

Vishal L. Handa[1], Bhoomi N. Patel[1], Ramesh K. Kothari[1], Dr. Arpita Bhattacharya[2], Dr. Ghanshyam Kavathiya[2], BRM Vyas[1]*

[1]Saurashtra University, (Department of Biosciences), Rajkot (Gujarat), India, 360005.

[2]Pandit Deendayal Upadhyay Medical College, (Department of Microbiology), Rajkot (Gujarat), India, 360001.

*Corresponding Author: BRM Vyas, ✉email: brmvyas@hotmail.com

Supplementary Tables

Table S1 Antibiotics class and abbreviation used in this study

Antibiotics			Concentration (μg/disc)
**Name**	**Class**	**Abbreviations**	
Tetracycline	Tetracycline	TE	30
Ciprofloxacin		CIP	5
Levofloxacin	Fluoroquinolones	LVX	5
Norfloxacin		NX	10
Meropenem		MEM	10
Imipenem-EDTA	Carbapenem	IE	10/750
Imipenem		IPM	10
Ceftazidime		CAZ	30
Cefotaxime		CTX	30
Cefepime		FEP	30
Cefuroxime		CXM	30
Ceftriaxone		CRO	30
Cefpodoxime	Cephalosporin	CPD	10
Cefixime		CFM	5
Cefoxitin		FOX	30
Cefatrizine		CFS	75
Ceftazidime-clavulanate		CAC	30/10
Ampicillin-sulbactam	β-lactam adjuvants	SAM	10/10
Piperacillin-tazobactam		TZP	100/10
Trimethoprim-sulfamethoxazole	Antifolates	SXT	25
Trimethoprim		TMP	5
Amikacin	Aminoglycosides	AN	30
Gentamycin		GM	30
Penicillin	Penicillin	P	10
Ampicillin		AM	20
Polymyxin B	Lipopeptides	PB	300
Colistin		CL	10
Erythromycin	Macrolide	E	15
Linezolid	Oxazolidinone	LZD	30
Rifamycin	Ansamycin	RIF	5

Clindamycin	Lincosamides	CM	2
Vancomycin	Glycopeptide	VA	30
Chloramphenicol	Phenolics	C	30
Novobiocin	Aminocoumarin	NB	5
Nitrofurantoin	Thiazolide	NIT	300
Aztreonam	Monobactam	ATM	30

Table S2 Antibiotic resistance in non-clinical bacterial strains surveyed from the published reports

Isolates	Sources (Isolates number)	Antibiotics resistance (%)																
		CIP	LVX	SXT	GM	TE	CAZ	AMP	IPM	E	LZD	P	FOX	CM	MEM	VA	C	References
Staphylococcus spp	Flying insects (12)	0	-	-	0	-	-	-	-	8	-	20	0	33	-	-	0	(Boiocchi et al., 2019)
	Cockroaches (5)	0	-	20	20	60	-	-	-	60	-	0	-	-	-	0	0	(Pai et al., 2005)
	Cockroach (21)	-	-	-	0	-	-	-	-	29	-	100	-	10	-	5	0	(Menasria et al., 2015)
	Food (284)	2	-	-	0	10	-	-	-	-	0	92	-	8	-	0	-	(Pesavento et al., 2007)
	Food (51)	-	-	-	0	51	-	-	-	19	-	92	12	20	-	2	-	(Achek et al., 2018)
P. aeruginosa	Environmental samples (44)	2	-	-	5	-	9	-	32	-	-	-	-	-	-	-	-	(Kaszab et al., 2021)
	Cockroaches (2)	0	-	-	0	-	-	-	0	-	-	-	-	-	-	-	-	(Pai et al., 2005)
	Cockroaches (22)	0	-	-	29	-	0	-	0	-	-	-	-	-	-	-	-	(Wannigama et al., 2014)
	Cockroaches (7)	0	-	-	0	-	0	-	100	-	-	-	-	-	-	-	-	(Menasria et al., 2015)
K. pneumoniae	Flying insects (7)	100	100	-	100	-	-	-	-	-	-	-	-	-	-	-	-	(Boiocchi et al., 2019)
	Cockroaches (2)	0	-	100	0	50	-	-	-	-	-	-	-	-	-	-	-	(Pai et al., 2005)
	Bovine area (23)	-	-	-	0	30	-	-	-	-	-	-	-	-	-	-	-	(Talbot et al., 1980)
	Drinking water	-	-	-	0	0	-	-	-	-	-	-	-	-	-	-	-	(Talbot et al., 1980)

	(27)																	
	Market vegetable (19)	-	-	-	0	0	-	-	-	-	-	-	-	-	-	-	-	(Talbot et al., 1980)
	Cockroaches (42)	0	-	95	24	52	-	-	-	-	-	-	-	-	-	-	-	(Wannigama et al., 2014)
E. coli	Flying insects (3)	0	0	-	0	-	-	-	-	-	-	-	-	-	-	-	-	(Boiocchi et al., 2019)
	Cockroaches (10)	0	-	20	100	80	0	-	-	-	-	-	-	-	-	-	-	(Pai et al., 2005)
	Cockroaches (36)	0	-	22	0	82	9	-	-	-	-	-	-	-	-	-	-	(Wannigama et al., 2014)
Enterobacter spp	Flying insects (8)	0	0	-	0	-	-	-	-	-	-	-	-	-	-	-	-	(Boiocchi et al., 2019)
	Cockroaches (21)	0	-	91	18	20	0	79	-	-	-	-	-	-	-	-	-	(Wannigama et al., 2014)

Supplementary Figures

(A)

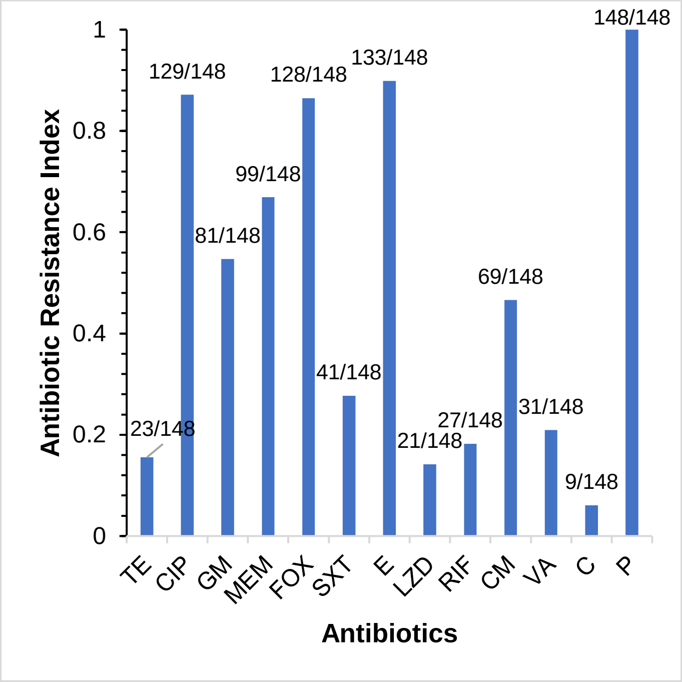

(B)

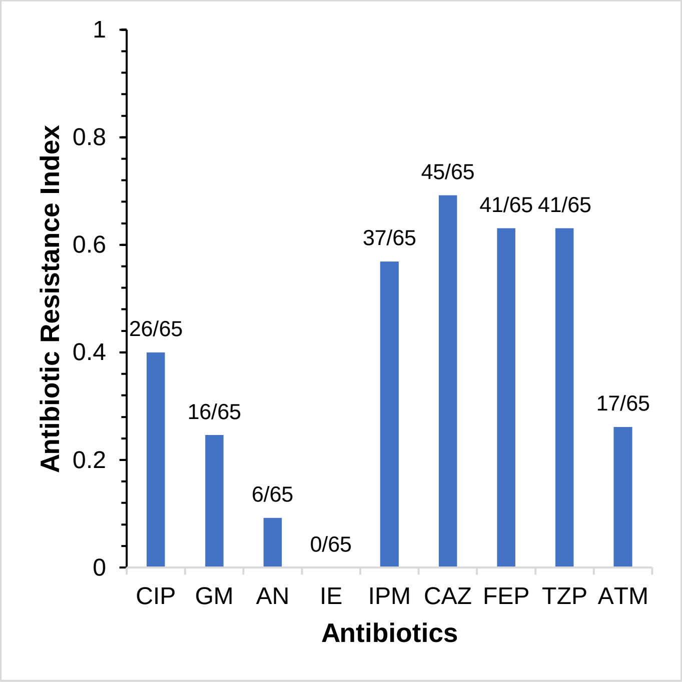

(C)

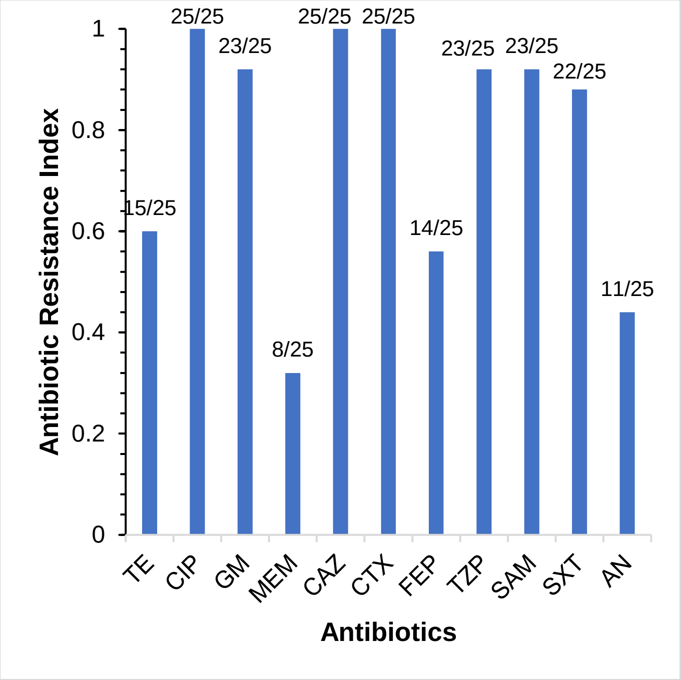
Antibiotic Resistance Index
1
0.8
0.6
0.4
0.2
0
15/25
25/25
23/25
8/25
25/25
25/25
14/25
23/25
23/25
22/25
11/25
TE
CIP
GM
MEM
CAZ
CTX
FEP
TZP
SAM
SXT
AN
Antibiotics

(D)

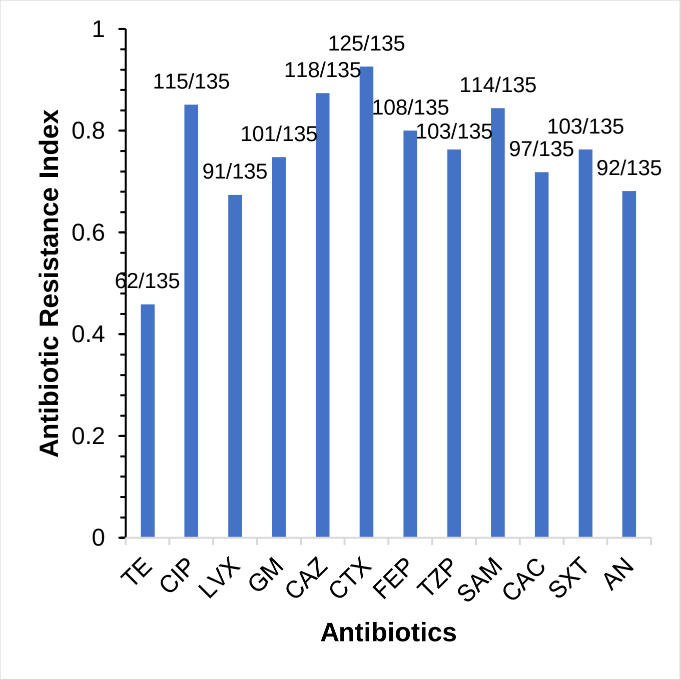
Antibiotic Resistance Index
1
0.8
0.6
0.4
0.2
0
62/135
115/135
91/135
101/135
118/135
125/135
108/135
103/135
114/135
97/135
103/135
92/135
TE
CIP
LVX
GM
CAZ
CTX
FEP
TZP
SAM
CAC
SXT
AN
Antibiotics

(E)

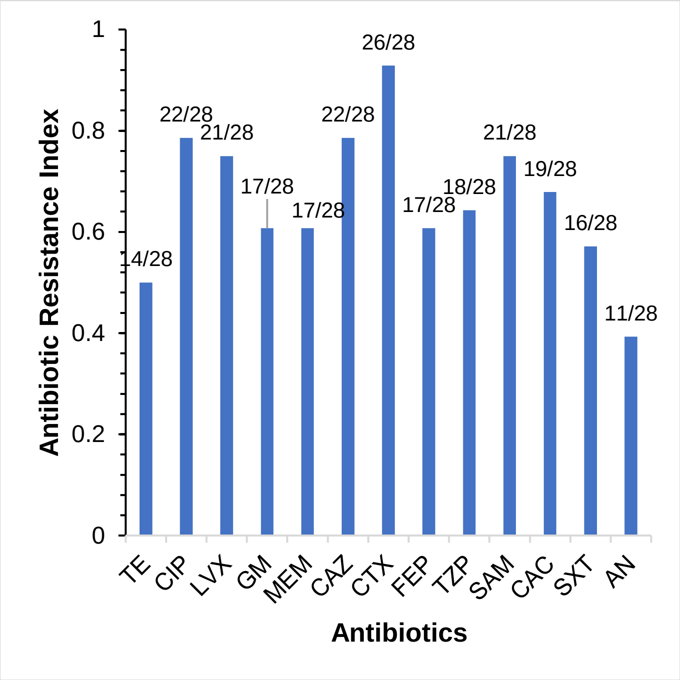

(F)

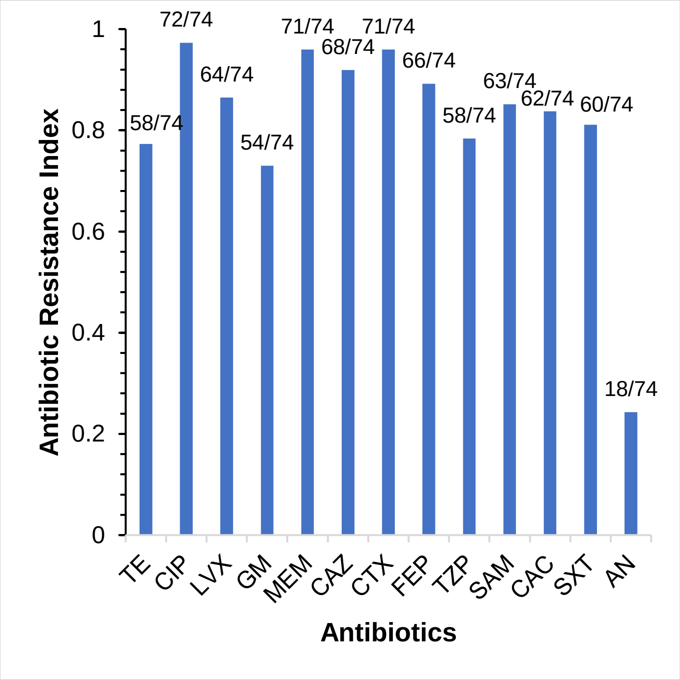

Figure S1 Antibiotic resistance percentage of each bacterial pathogens. Antibiotics were tested >90% against each bacterial pathogens to determine the Antibiotic resistance (R_I), (resistance incidence/total collected pathogens), (A) *Staphylococcus* spp, (B) *Pseudomonas aeruginosa*, (C) *Acinetobacter* spp, (D) *Klebsiella pneumoniae*, (E) *Enterobacter* spp, (F) *Escherichia coli*.

(A) Selection pattern for *K. pneumoniae*

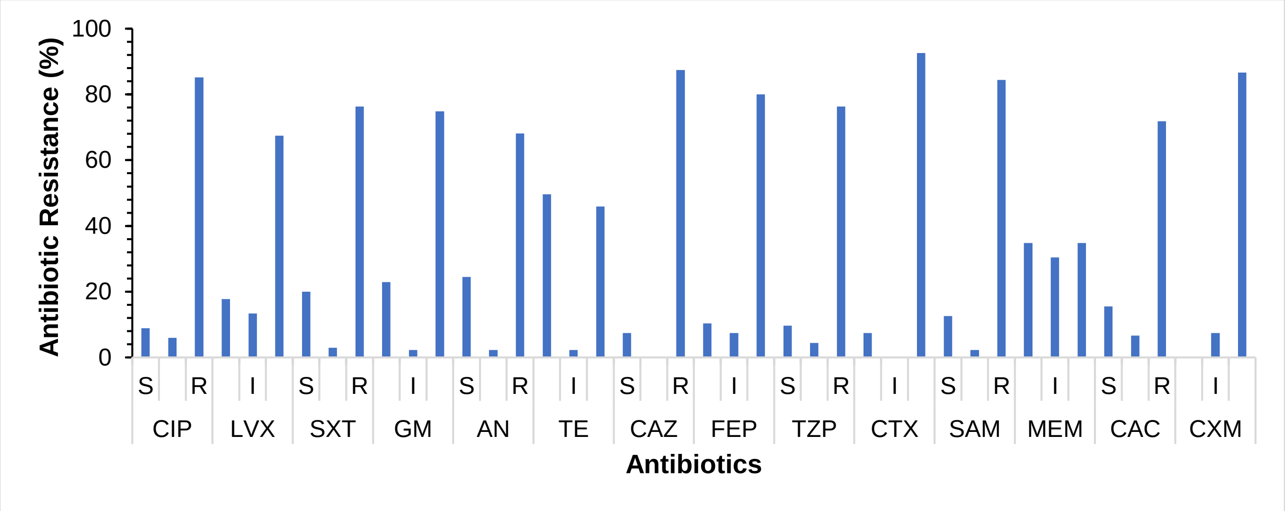

(B) Selection pattern for *E. coli*

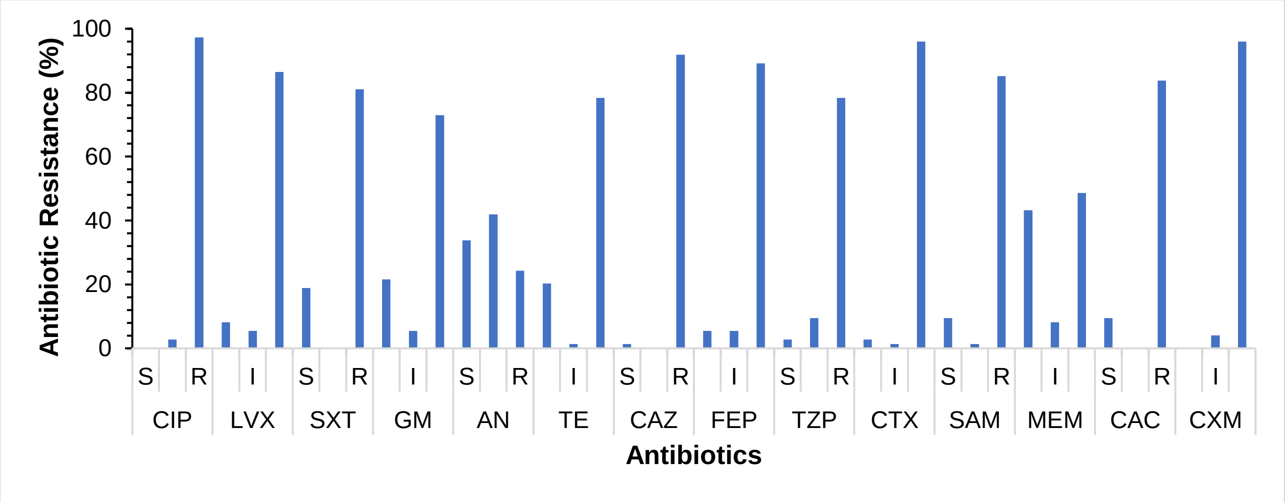

(C) Selection pattern for *P. aeruginosa*

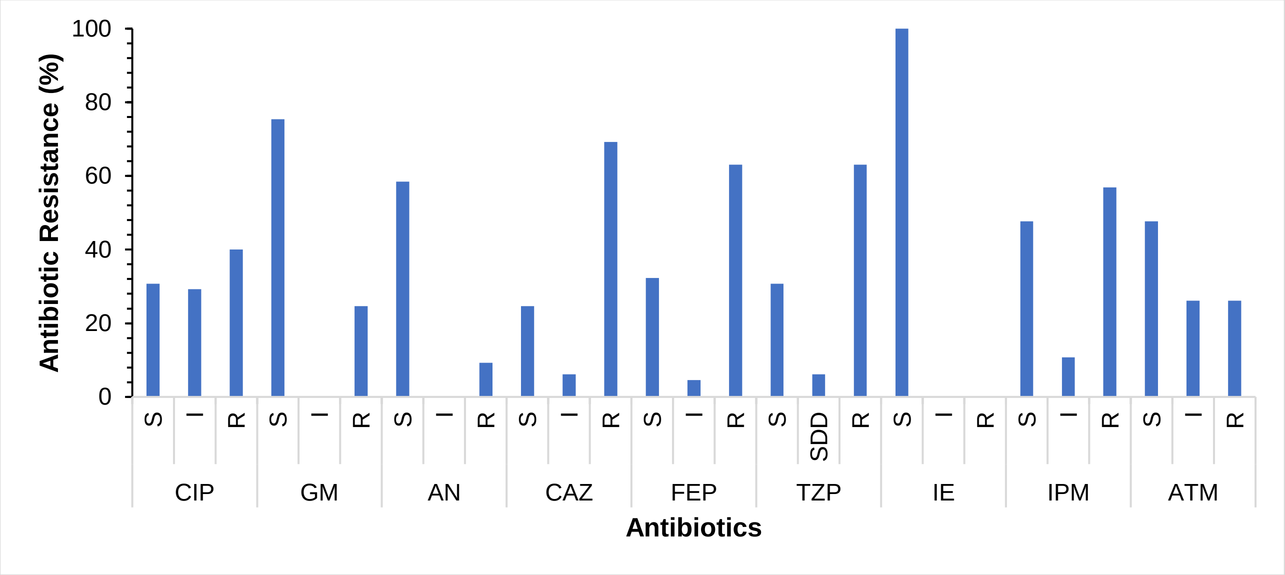

(D) Selection pattern for *Staphylococcus* species

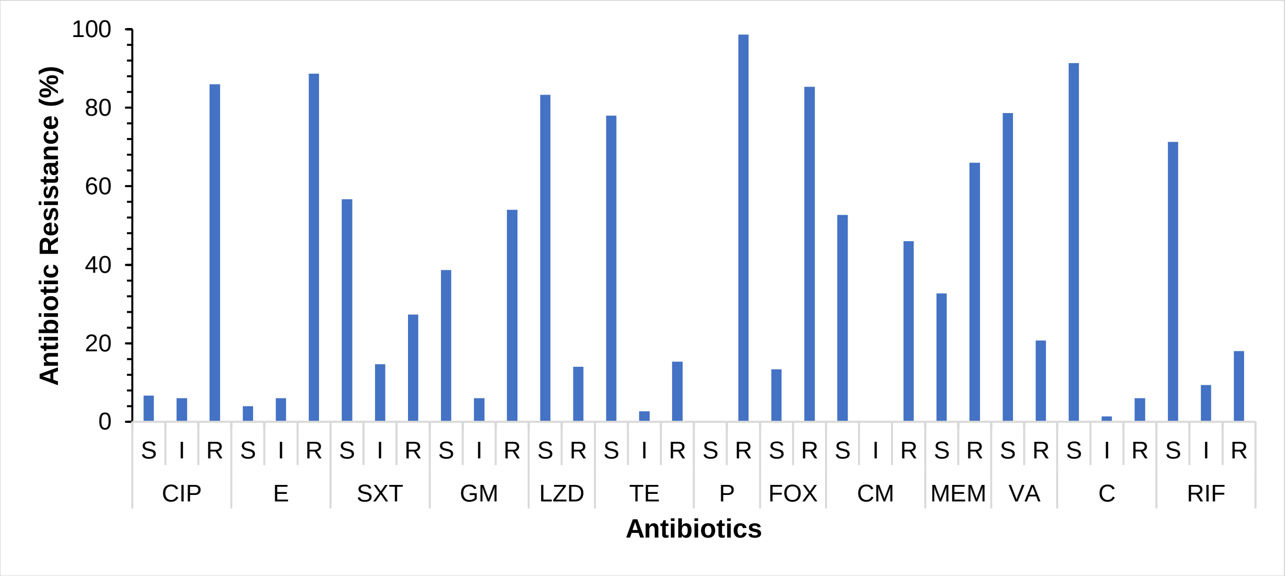

(E) Selection pattern for isolates *Enterobacter*

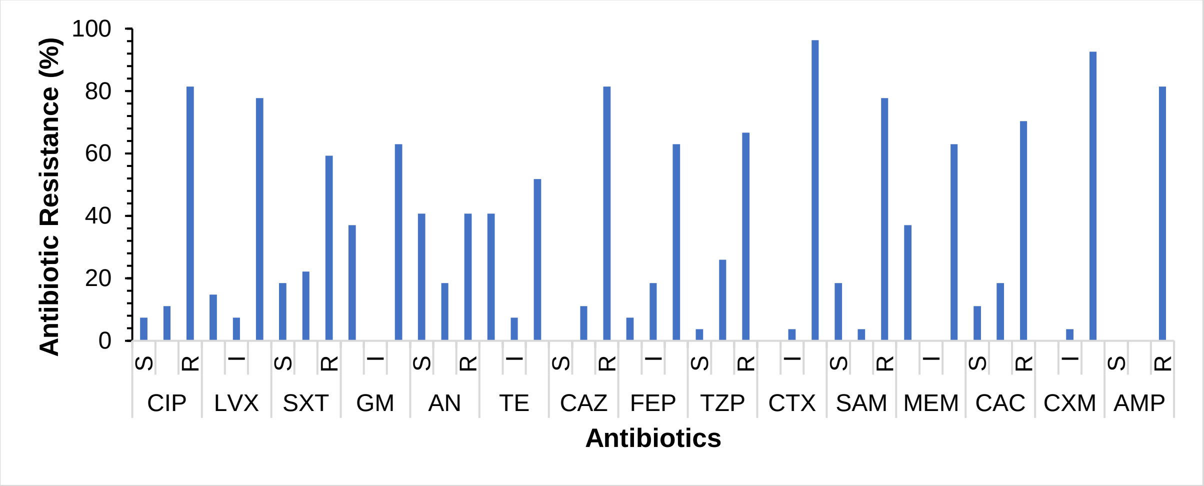

(F) Selection pattern for isolates *Acinetobacter* species

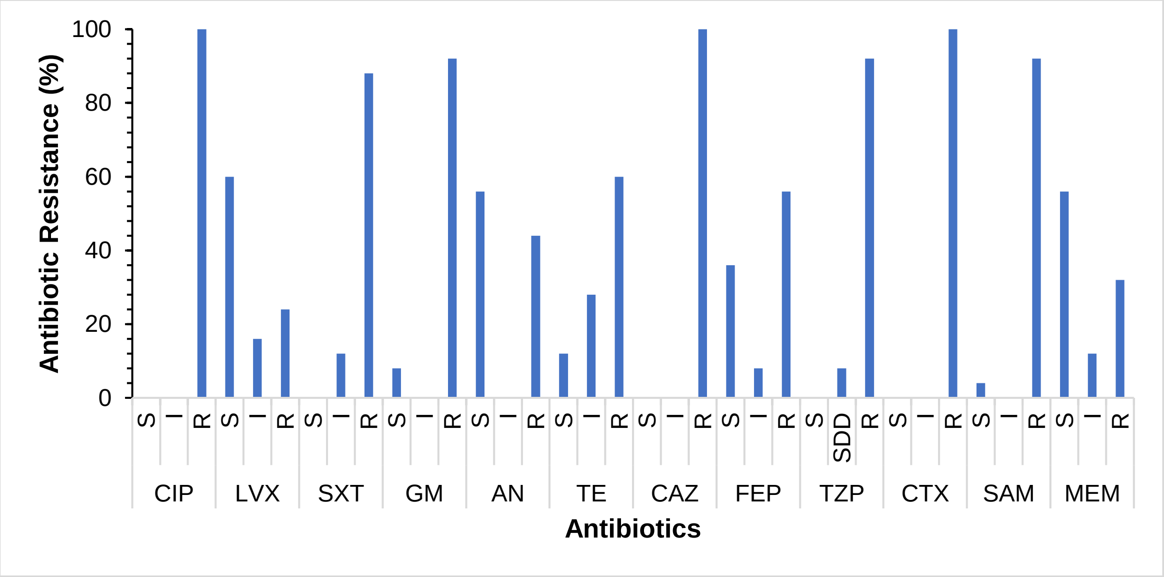

Figure S2 Selection pattern based on antibiotic susceptibility phenotypic traits.

References

Achek, R., Hotzel, H., Cantekin, Z., Nabi, I., Hamdi, T. M., Neubauer, H., et al., (2018). Emerging of antimicrobial resistance in staphylococci isolated from clinical and food samples in Algeria. *BMC research notes*, *11*, 1-7. https://doi.org/10.1186/s13104-018-3762-2 (2018).

Boiocchi, F., Davies, M. P., & Hilton, A. C. (2019). An examination of flying insects in seven hospitals in the United Kingdom and carriage of bacteria by true flies (Diptera: Calliphoridae, Dolichopodidae, Fanniidae, Muscidae, Phoridae, Psychodidae, Sphaeroceridae). *Journal of medical entomology*, *56*, 1684-1697. https://doi.org/10.1093/jme/tjz086.

Kaszab, E., Radó, J., Kriszt, B., Pászti, J., Lesinszki, V., Szabó, A., et al., (2021). Groundwater, soil and compost, as possible sources of virulent and antibiotic-resistant

Pseudomonas aeruginosa. *International Journal of Environmental Health Research*, *31*, 848-860. doi: 10.1080/09603123.2019.1691719.

Menasria, T., Samir, T. I. N. E., Mahcene, D., Benammar, L., Megri, R., Boukoucha, M., et al., (2015). External bacterial flora and antimicrobial susceptibility patterns of *Staphylococcus* spp. and Pseudomonas spp. isolated from two household cockroaches, *Blattella germanica* and *Blatta orientalis*. *Biomedical and Environmental Sciences*, *28*, 316-320. https://doi.org/10.3967/bes2015.045.

Pai, H. H., Chen, W. C., & Peng, C. F. (2005). Isolation of bacteria with antibiotic resistance from household cockroaches (*Periplaneta americana* and *Blattella germanica*). *Acta tropica*, *93*, 259-265. https://doi.org/10.1016/j.actatropica.2004.11.006.

Pesavento, G., Ducci, B., Comodo, N., & Nostro, A. L. (2007). Antimicrobial resistance profile of *Staphylococcus aureus* isolated from raw meat: A research for methicillin resistant *Staphylococcus aureus* (MRSA). *Food control*, *18,* 196-200. https://doi.org/10.1016/j.foodcont.2005.09.013.

Talbot Jr, H. W., Yamamoto, D. K., Smith, M. W., & Seidler, R. J. (1980). Antibiotic resistance and its transfer among clinical and nonclinical *Klebsiella* strains in botanical environments. *Applied and Environmental Microbiology*, *39,* 97-104. https://doi.org/10.1128/aem.39.1.97-104.1980.

Wannigama, D. L., Dwivedi, R., & Zahraei-Ramazani, A. (2014). Prevalence and antibiotic resistance of gram-negative pathogenic bacteria species isolated from *Periplaneta americana* and *Blattella germanica* in Varanasi, India. *Journal of Arthropod-Borne Diseases*, *8*, 10.
